# Messenger RNA Oxidation Occurs Early in Disease Pathogenesis and Promotes Motor Neuron Degeneration in ALS

**DOI:** 10.1371/journal.pone.0002849

**Published:** 2008-08-06

**Authors:** Yueming Chang, Qiongman Kong, Xiu Shan, Guilian Tian, Hristelina Ilieva, Don W. Cleveland, Jeffrey D. Rothstein, David R. Borchelt, Philip C. Wong, Chien-liang Glenn Lin

**Affiliations:** 1 Department of Neuroscience, The Ohio State University, Columbus, Ohio, United States of America; 2 Ohio State Biochemistry Program, The Ohio State University, Columbus, Ohio, United States of America; 3 Department of Medicine and Neuroscience, University of California San Diego, San Diego, California, United States of America; 4 Department of Neurology, John Hopkins University, Baltimore, Maryland, United States of America; 5 Department of Neuroscience, University of Florida, Gainesville, Florida, United States of America; 6 Department of Pathology, John Hopkins University, Baltimore, Maryland, United States of America; National Institutes of Health, United States of America

## Abstract

**Background:**

Accumulating evidence indicates that RNA oxidation is involved in a wide variety of neurological diseases and may be associated with neuronal deterioration during the process of neurodegeneration. However, previous studies were done in postmortem tissues or cultured neurons. Here, we used transgenic mice to demonstrate the role of RNA oxidation in the process of neurodegeneration.

**Methodology/Principal Findings:**

We demonstrated that messenger RNA (mRNA) oxidation is a common feature in amyotrophic lateral sclerosis (ALS) patients as well as in many different transgenic mice expressing familial ALS-linked mutant copper-zinc superoxide dismutase (SOD1). In mutant SOD1 mice, increased mRNA oxidation primarily occurs in the motor neurons and oligodendrocytes of the spinal cord at an early, pre-symptomatic stage. Identification of oxidized mRNA species revealed that some species are more vulnerable to oxidative damage, and importantly, many oxidized mRNA species have been implicated in the pathogenesis of ALS. Oxidative modification of mRNA causes reduced protein expression. Reduced mRNA oxidation by vitamin E restores protein expression and partially protects motor neurons.

**Conclusion/Significance:**

These findings suggest that mRNA oxidation is an early event associated with motor neuron deterioration in ALS, and may be also a common early event preceding neuron degeneration in other neurological diseases.

## Introduction

Oxidative damage is believed to be an important contributor to the pathogenesis of many neurodegenerative diseases including amyotrophic lateral sclerosis (ALS) [1]. It results from modification of vital cellular components, including proteins, lipids and nucleic acids, by free radicals. Reactive oxygen species can hydroxylate guanine to produce 8-oxo-7,8-dihydroguanosine (8-OHG) in RNA, the most abundant oxidized base [2], [3]. Nunomura *et al.* [4] showed that most of the oxidized nucleosides are associated with cytoplasmic RNA and are restricted to vulnerable neurons in the brains of Alzheimer's disease (AD). Similar RNA oxidation in neuronal cytoplasm was also observed in other disorders [5]–[10]. We previously demonstrated that up to 50% of messenger RNA (mRNA) are oxidized in AD frontal cortices [11]. Some mRNA species are more susceptible to oxidative damage, and many of them have been implicated in the pathogenesis of AD [12]. Oxidized mRNA cannot be translated properly leading to reduced protein expression and consequently, loss of normal protein function [12]–[14]. Ribosomal RNA oxidation was also reported in AD brains [15], [16]. There is a considerable amount of evidence supporting an early involvement of RNA oxidation in the pathological cascade of neurodegeneration [4], [13], [15], [17], [18].

ALS is a fatal neurodegenerative disorder that is characterized by progressive degeneration of motor neurons in the spinal cord, motor cortex and brainstem [19]. The majority of cases have no genetic component, i.e. sporadic ALS (SALS). Approximately 5% of ALS cases are familial (FALS), usually with an autosomal dominant inheritance pattern. About 15–25% of FALS cases are linked to mutation in the gene encoding the antioxidant enzyme Cu^2+^/Zn^2+^ superoxide dismutase (SOD1). It is believed that multiple mechanisms underlie the disease progression [19]. Oxidative stress is considered to be one of the important factors [1].

Over-expression of some of FALS-linked mutant SOD1 proteins in transgenic mice results in the development of a neurological disorder that resembles ALS [20]. Mutant SOD1 causes motor neuron degeneration by acquiring a toxic gain of function property rather than by the loss of enzymatic activity [21]. Many studies have shown that the mutant SOD1 toxicity to motor neurons is non-cell autonomous, i.e. mutant damage is required within both motor neurons and non-neuronal cells [22]. In the present study, we investigated whether RNA oxidation plays a role in the pathogenesis of motor neuron degeneration. Our data indicate that RNA oxidation is a common feature in ALS and that RNA oxidation may occur early in disease pathogenesis and promote motor neuron deterioration in ALS.

## Results

### Poly(A)^+^ mRNAs isolated from ALS-affected areas are oxidized

**Figure 1:**
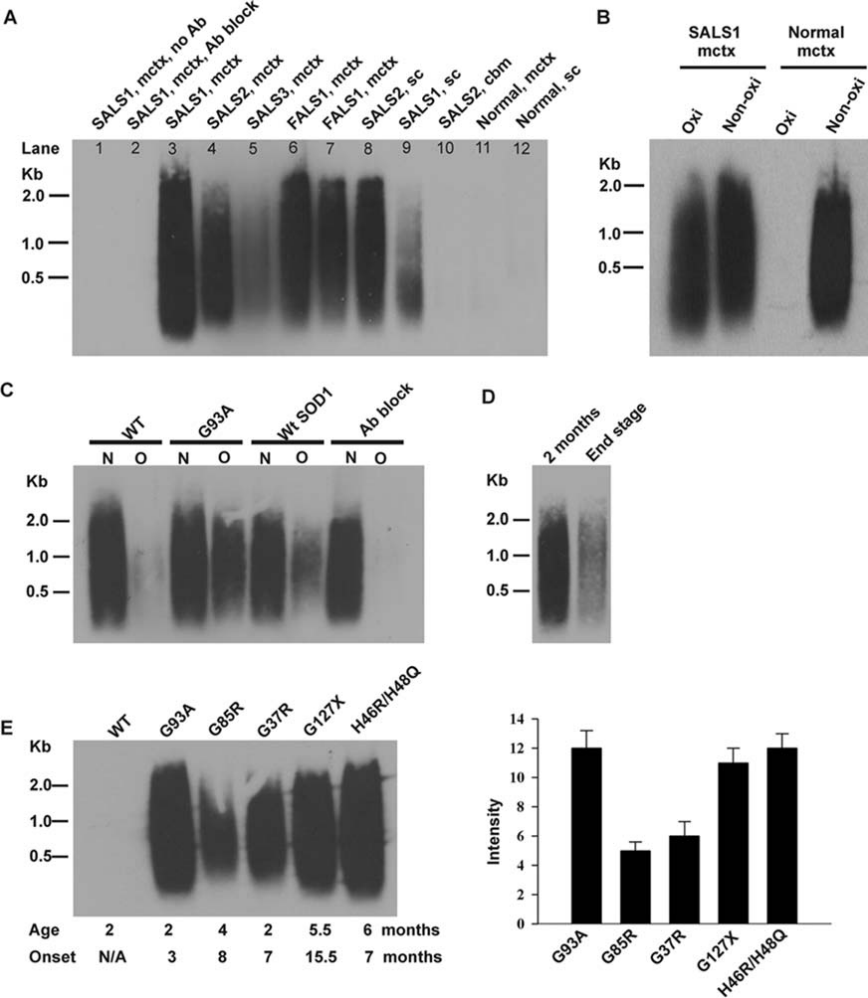
mRNAs are oxidatively damaged in ALS-affected areas and pre-symptomatic stage of mutant SOD1 mice. (A) Southern blot analysis of 15A3-immunoprecipitated oxidized mRNAs in ALS patients. mctx, motor cortex; sc, spinal cord; cbm, cerebellum; no Ab, no antibody; Ab block, antibody block (antibodies pre-incubated with 8-OHG). (B) Quantitative analysis of the magnitude of mRNA oxidation by comparing the signal density of oxidized mRNAs (*Oxi*) to serial dilutions of non-oxidized mRNA (*Non-oxi*) via Southern blot analysis. An example of analysis of SALS1 motor cortex is shown. The non-oxidized mRNA fraction was diluted 1 to 15. (C) Southern blot analysis of oxidized (*O*) and non-oxidized (*N*) mRNA pools prepared from spinal cords of indicated mice (60 day-old, n = 3). The percentages of total spinal cord mRNA that were oxidized in SOD1^G93A^ (*G93A*), SOD1^WT^ (*WtSOD1*), and non-transgenic (*WT*) mice are about 30%, 3–5%, and barely detectable, respectively. No mRNA was precipitated in the SOD1^G93A^ sample when 15A3 antibodies were pre-incubated with 8-OHG (*Ab block*). (D) Less amount of spinal cord mRNAs were oxidized (∼10% or less) in the end stage of SOD1^G93A^ mice (n = 3?). (E) RNA oxidation occurred in the pre-symptomatic stage of different mutant SOD1 mice. Oxidized mRNA pools were prepared from the spinal cords of indicated mutant SOD1 mice (n = 3). The ages of tested mice (*Age*) and the age of onset (*Onset*) are noted. Right panel shows densitometry analysis of Southern blot results.

We previously developed an immunoprecipitation (IP) procedure using the monoclonal antibody 15A3, which recognizes 8-OHG in the oxidized RNA, to isolate oxidized RNA [12]. We used this established procedure to investigate whether mRNAs are oxidized in ALS postmortem tissues. The following postmortem tissues were examined: 11 SALS motor cortices, 10 SALS lumbar spinal cords, 2 FALS motor cortices (without SOD1 mutations), 5 SALS cerebellums, 5 age-matched normal motor cortices, and 1 age-matched normal lumbar spinal cord. Poly(A)^+^ mRNAs were isolated from each tissue, and oxidized mRNAs were separated from non-oxidized mRNAs by IP with 15A3 antibodies. The isolated oxidized mRNAs were then reverse transcribed to cDNAs with DIG-labeled dUTPs to facilitate analysis by Southern blotting. Among 11 SALS motor cortex samples, 3 samples showed strong signals on the Southern blot such as SALS1 (Fig. 1A, lane 3), 5 samples showed moderate signals such as SALS2 (lane 4), and 3 samples showed weak signals such as SALS3 (lane 5). Both FALS motor cortex samples showed moderate signals (lane 6 and 7). Among 10 SALS spinal cord samples, 6 samples showed moderate signals such as SALS2 (lane 8), and 4 samples showed very weak signals such as SALS1 (lane 9). There was no or very weak signals in ALS cerebellum (lane 10), normal motor cortex (lane 11) or normal spinal cord (lane 12). A no-antibody control (lane 1) and an 8-OHG-blocked antibody control (lane 2) were also carried out to confirm that the precipitated mRNAs were oxidized mRNAs. The increased mRNA oxidation in ALS tissues did not result from agonal state of disease because it was not observed in the unaffected areas, cerebellum. Further, the postmortem interval did not affect the amount of immunoprecipitated mRNAs. These results indicated that mRNAs are oxidatively damaged to a variable extent in ALS patients.

We quantified the magnitude of mRNA oxidation by comparing the signal densities of oxidized mRNAs (immunoprecipitated RNAs) to serial dilutions of non-oxidized mRNA (unprecipitated RNAs) via Southern blot analysis. Quantitative analysis revealed that about 6–10% of the mRNAs were oxidized in ALS tissues showing strong signals on the Figure 1A Southern blot such as the SALS1 motor cortex (Fig. 1B), and about 3–5% of the mRNAs were oxidized in ALS tissues showing moderate signals.

### Significant amounts of poly(A)^+^ mRNAs are oxidized in the spinal cords of mutant SOD1 mice at the pre-symptomatic stage

We investigated whether mRNA oxidation occurs in transgenic mice expressing FALS-linked mutant SOD1. We first examined mRNA prepared from the spinal cords of 60 day-old (pre-symptomatic stage) SOD1^G93A^ mice. Non-transgenic littermates and age-match transgenic mice over-expressing wild-type SOD1 (SOD1^WT^) were used as controls (n = 3 per group). Oxidized mRNAs (*O*) were separated from non-oxidized mRNAs (*N*) by IP with 15A3 antibodies and then analyzed by Southern blotting. Approximately 30% and 4% of total spinal cord mRNAs were oxidized in SOD1^G93A^ (Fig. 1C, *G93A*) and SOD1^WT^ (Fig. 1C, *WtSOD1*) spinal cords, respectively, while a trace amount of mRNA was oxidized in non-transgenic spinal cords (Fig. 1C, *WT*). These results indicated a significant oxidative damage of mRNAs in the spinal cords of SOD1^G93A^ mice at an early, pre-symptomatic stage. We further examined oxidized mRNA level in the end stage of SOD1^G93A^ spinal cords. The results showed that much lower amounts of total spinal cord mRNAs were oxidized (∼10% or less) in the end stage compared to the pre-symptomatic stage (Fig. 1D), which was consistent with the above ALS postmortem tissues study (Fig. 1A).

Furthermore, we examined whether mRNA oxidation occurs in other mutant SOD1 transgenic mice at pre-symptomatic stage, including SOD1^G37R^ (2 month-old), SOD1^G85R^ (4 month-old), SOD1^G127X^ (5.5 month-old), and SOD1^His46R/His48Q^ (6 month-old) (n = 3 per group). These mice develop the disease onsets at age of ∼7–15.5 months. Southern blot analysis of 15A3-immunoprecipitated mRNAs prepared from the spinal cords showed similar levels of oxidized mRNA in SOD1^G127X^ and SOD1^His46R/His48Q^ but less in SOD1^G37R^ and SOD1^G85R^ compared to SOD1^G93A^ (Fig. 1E). These results suggest that mRNA oxidation may be a common early event preceding motor neuron degeneration in ALS.

### A significant increase in RNA oxidation in the motor neurons and oligodendrocytes of the SOD1^G93A^ spinal cord at pre-symptomatic stage

**Figure 2:**
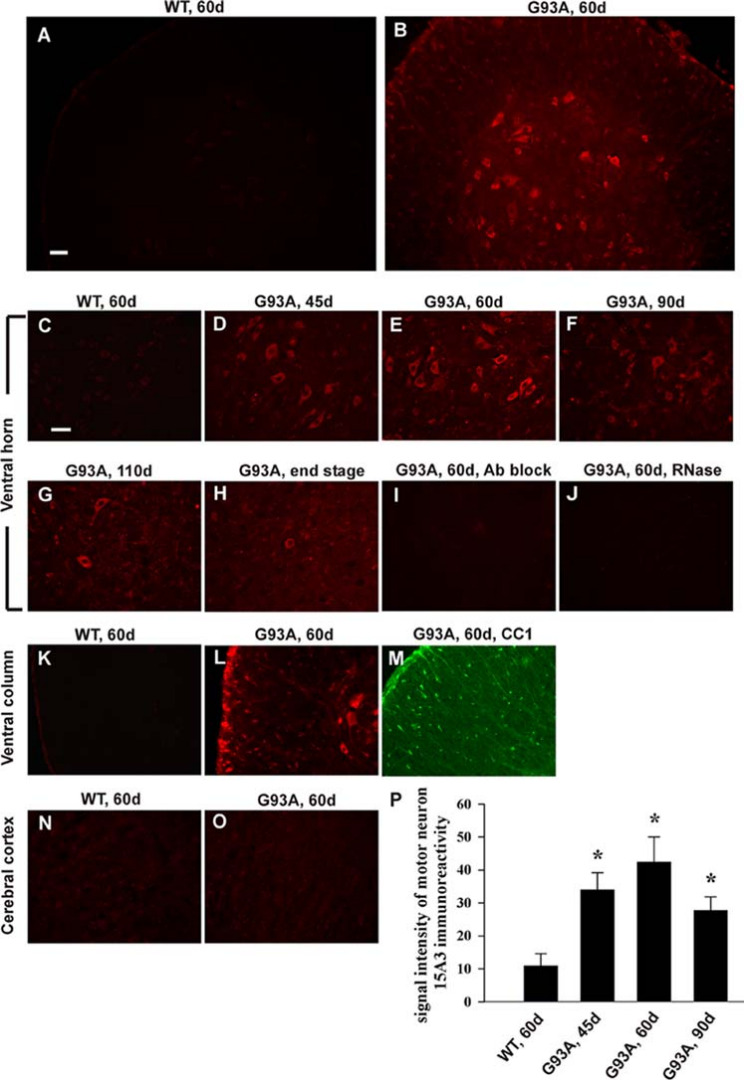
Increased RNA oxidation in motor neurons and oligodendrocytes of SOD1^G93A^ spinal cord at pre-symptomatic stage. Lumbar spinal cord sections from different ages of SOD1^G93A^ mice (*G93A*) and non-transgenic littermates (*WT*) were immunolabeled with 15A3 antibodies. The intensity of 15A3 immunofluorescence in SOD1^G93A^ mice was significantly increased at 45 days of age (D), further enhanced at 60 days of age (B, E), and then diminishes during the symptomatic stage (F, G, H), while only a very faint signal was detected in non-transgenic littermates (A, C). 15A3 immunoreactivity was prominent in the ventral horn motor neurons and the white matter oligodendrocytes (K, L), as identified by CC1 immunostaining (M). No increase of 15A3 immunofluorescence was observed in the brain sections (N, O). The immunoreactivity was diminished greatly by the RNase treatment (J) and when the antibody was pre-incubated with 8-OHG (I). Scale bar, 50 µm. (P) Statistical analysis of motor neuron 15A3 immunofluorescence intensity (n = 20). Asterisks indicate statistical significance compared with WT, 60 d (*P<0.0001).

To better understand at which stage RNA oxidation occurs and whether RNA oxidation plays a role in the pathogenesis of motor neuron degeneration, we further investigated RNA oxidation in SOD1^G93A^ mice. We first examined what cell types showed increased RNA oxidation in the lumbar spinal cord from different ages of SOD1^G93A^ mice or non-transgenic littermates (n = 3 per group). Immunofluorescent staining was performed using the 15A3 antibody, which recognizes both 8-OHG and 8-OHdG, markers of oxidative damage to RNA and DNA respectively. The intensity of 15A3 immunofluorescence was significantly increased in 45 day-old SOD1^G93A^ mice (Fig. 2D) and further enhanced in 60 day-old SOD1^G93A^ mice (Fig. 2B&E), while only a very faint signal was detected in non-transgenic littermates (Fig. 2A&C). 15A3 immunoreactivity was prominent in the ventral horn motor neurons as judged by their characteristic size and morphology. In addition, a significant increase of 15A3 immunoreactivity was also observed in the white matter oligodendrocytes (Fig. 2K&L) as identified by CC1 immunostaining (Fig. 2M). Importantly, 15A3 signal intensity was prominent in the cytoplasm and was diminished greatly by the RNase treatment (Fig. 2J) but only slightly by DNase I treatment, which indicated that cytoplasmic RNA was the major site of nucleic acid oxidative damage. Moreover, the immunoreactivity was diminished completely when the antibody was pre-incubated with 8-OHG (Fig. 2I), indicating that the observed fluorescent signal was specific to oxidized base 8-OHG in RNA. During the symptomatic stage, 15A3 signal intensity was decreased in 90 day-old SOD1^G93A^ mice (Fig. 2F), and further reduced in 110 day-old SOD1^G93A^ mice (Fig. 2G). The 15A3 immunoreactivity was low in the dying motor neurons, and started to appear in the glial cells, which was significant at the end stage (Fig. 2H). Figure 2P shows the statistical analysis of motor neuron 15A3 immunofluorescence intensity. We also examined brain sections from 60 day and 90 day-old SOD1^G93A^ mice as well as non-transgenic littermates. No increase in 15A3 immunofluorescence was observed (Fig. 2N&O). These results indicated that RNA oxidation occurs as early as 45 days of age, progressively increasing with age until it peaks at 60–70 days of age (when the motor neurons still look healthy) and then diminishes when the motor neurons begin to degenerate.

### RNA oxidation is an early event far preceding motor neuron degeneration

**Figure 3:**
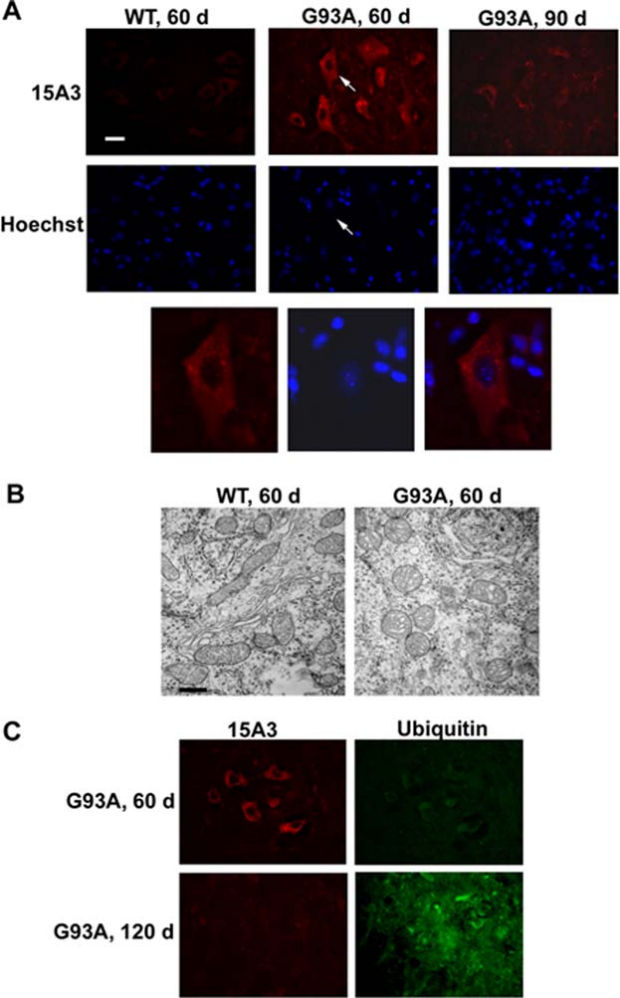
RNA oxidation is an early event preceding motor neuron degeneration. Lumbar spinal cords from indicated age of SOD1^G93A^ mice (*G93A*) and non-transgenic littermates (*WT*) were examined. (A) In the ventral horn, motor neurons with strong 15A3 immunoreactivity had normal nuclear and chromatin morphology by Hoechst 33342 staining; the dying motor neurons showed abnormal nuclear and chromatin morphology but less 15A3 immunoreactivity. *Arrows* point to the same neuron. Scale bar, 25 µm. (B) Motor neurons with strong 15A3 immunoreactivity had only minor mitochondrial vacuolization as examined by electron microscopy. Scale bar, 0.5 µm. (C) Motor neurons with strong 15A3 immunoreactivity did not have ubiquitinated protein aggregation.

To further examine those motor neurons showing strong 15A3 immunoreactivity, we performed nuclear staining using chromatin-binding dye bis-benzimide (Hoechst 33342). The results showed that motor neurons with strong 15A3 signal had normal nuclear and chromatin morphology (Fig. 3A, *G93A 60d*). At the symptomatic stage (90 days), the dying motor neurons showed abnormal nuclear and chromatin morphology but less 15A3 immunoreactivity (Fig. 3A, *G93A 90d*). Mitochondrial vacuolization is considered to be one of the earliest molecular pathological changes in SOD1^G93A^ mice. We examined mitochondrial morphology by electron microscopy, and the results showed that motor neurons with strong 15A3 immunoreactivity had only minor mitochondrial vacuolization (Fig. 3B). Ubiquitinated protein aggregation is a hallmark of degenerating motor neurons. We did not observe ubiquitinated protein aggregation in motor neurons with strong 15A3 immunoreactivity at age of 60 days (Fig. 3C). These results indicate that those motor neurons showing RNA oxidation still appear to be healthy, and that RNA oxidation is an early event.

### Some mRNA species are more susceptible to oxidative damage

**Figure 4:**
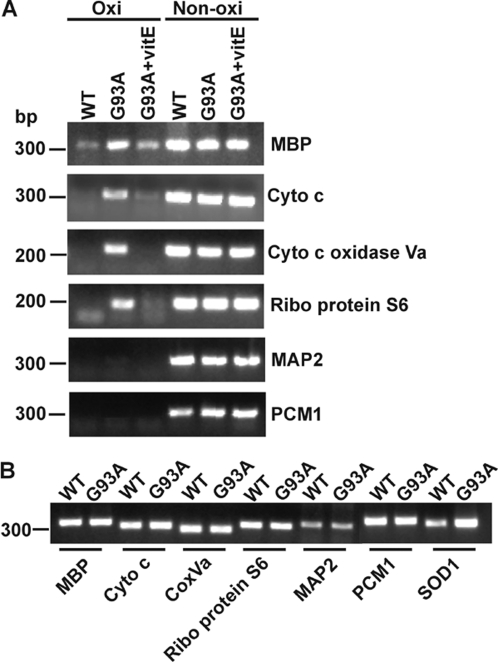
Some mRNA species are more susceptible to oxidative damage in SOD1^G93A^ mice. (A) Semi-quantitative RT-PCR analysis confirmed that the identified mRNA species by DNA microarray were present in the oxidized mRNA pool. MBP, cytochrome c, cytochrome c oxidase Va and ribosome protein S6 mRNAs, which had strong signal intensities on the arrays, were present in the oxidized mRNA pool. MAP2 and PCM1 mRNAs, which had very weak signal intensities on the arrays, were hardly detected in the oxidized mRNA pool. These oxidized mRNA species are significantly decreased in vitamin E treated SOD1^G93A^ mice (*G93A+vitE*). (B) Semi-quantitative RT-PCR analysis showed that the oxidized mRNA species are not upregulated in the whole spinal cord of SOD1^G93A^ mice compared to their non-transgenic littermates. SOD1 mRNA, which includes endogenous mouse SOD1 and transgenic human SOD1, was used as a control. n = 3.

To identify oxidized mRNA species, we performed DNA microarrays using Affymetrix GeneChip Mouse Genome 430 2.0. This array can analyze ∼39,000 transcripts. Three arrays were performed, and in each array, oxidized mRNAs were prepared from two spinal cords of 60-day old SOD1^G93A^ mice so a total of six mice were analyzed. A total of 3,409 mRNA species were identified, and these oxidized mRNA species and their relative signal intensities on the chips were similar among the three arrays. Semi-quantitative PCR analysis was then performed to verify that the identified mRNA species were present in the oxidized mRNA pool. As shown in Figure 4A, ribosome protein S6, cytochrome c oxidase Va, cytochrome c and myelin basic protein (MBP) mRNAs, which had strong signal intensities on the arrays, were present in the oxidized mRNA pool. Microtubule-associated protein 2 (MAP2) and pericentriolar material 1 (PCM1) mRNAs, which had very weak signal intensities on the arrays, were hardly detected in the oxidized mRNA pool. The signal intensities of PCR products were quite consistent with the signal intensities of the oxidized mRNA species on the arrays.

We then used the computer program Onto-Express (http://vortex.cs.wayne.edu/projects.htm#Onto-Express) to analyze the identified oxidized mRNA species and grouped them according to cellular component or molecular function [23]. The analysis based on the cellular component showed that mRNA species encoded for proteins that localized to mitochondria, ribosome and cytosol were more susceptible to oxidation. The analysis based on the molecular function showed that mRNA species encoded for proteins that are involved in mitochondrial electron transport, protein biosynthesis, myelination, protein folding and degradation, cytoskeleton, tricarboxylic acid cycle and glycolysis, were more susceptible to oxidation.

The signal intensities of the oxidized mRNA species on the chips ranged from ∼200 to ∼12,000. There were 60 mRNA species whose signal intensity was above 5,000, 298 mRNA species whose signal intensity was between 2,000 and 5,000, 648 mRNA species whose signal intensity was between 1,000 and 2,000, and 2,403 mRNA species whose signal intensity was below 1,000. We considered those mRNA species, whose signal intensity was above 2,000, were highly oxidized. Table 1 shows the list of these mRNA species grouped by their function (uncharacterized genes are not listed). Importantly, some of these oxidized mRNAs appear to be related to the pathogenesis of ALS. Several highly oxidized mRNA species correspond to genes linked to familial ALS or ALS-like human motor neuron disease, including SOD1, dynactin 1, and vesicle-associated membrane protein 1.

**Table 1:** List of highly oxidized mRNA species in the spinal cord of 60 day-old SOD1^G93A^ mice.

Gene accession	Gene title	Intensity index*		SD
**protein biosynthesis**				
AK013880	asparaginyl-tRNA synthetase	2.2	±	0.8
BQ176989	eukaryote translation initiation factor 5	1.6	±	0.6
BC024899	eukaryote translation initiation factor 5a	1.6	±	0.8
BI693609	eukaryotic translation elongation factor 1	2.3	±	0.7
BC018223	eukaryotic translation elongation factor 1 alpha 1	1.5	±	1.0
NM_017404	mitochondrial ribosomal protein L39	3.9	±	3.9
NM_052835	ribosomal protein L10	1.5	±	0.5
AI506565	ribosomal protein L13	2.4	±	0.6
AI324936	ribosomal protein L13a	5.8	±	0.3
NM_001002239	ribosomal protein L17	3.5	±	1.0
AK008457	ribosomal protein L21	1.5	±	0.7
AV124394	ribosomal protein L23	6.9	±	0.7
BC002110	ribosomal protein L24	2.0	±	0.6
NM_013762	ribosomal protein L3	3.4	±	0.8
NM_009083	ribosomal protein L30	2.6	±	0.6
AV109882	ribosomal protein L31	5.8	±	1.1
BM114165	ribosomal protein L5	3.0	±	1.1
NM_011292	ribosomal protein L9	1.5	±	0.5
AI413680	ribosomal protein S12	2.4	±	0.9
BB724547	ribosomal protein S17	2.6	±	0.5
AV046829	ribosomal protein S20	3.2	±	1.1
NM_024277	ribosomal protein S27a	3.8	±	2.2
BC010987	ribosomal protein S28	4.6	±	0.7
AV037157	ribosomal protein S29	4.8	±	0.6
AW108231	ribosomal protein S3	2.6	±	0.6
NM_009096	ribosomal protein S6	4.7	±	0.7
NM_011300	ribosomal protein S7	2.4	±	0.4
NM_019883	ubiquitin A-52 residue ribosomal protein fusion product 1	4.6	±	0.6
**Cytoskeleton**				
NM_013798	actin-like (Actl)	3.0	±	1.3
BB758476	actin-related protein 3 homolog	1.5	±	0.6
AV148266	cofilin 1, non-muscle	5.8	±	1.5
NM_011722	dynactin 6	2.2	±	0.8
NM_019682.1	dynein, cytoplasmic, light peptide	2.2	±	0.8
AV224521	gelsolin	4.1	±	0.5
AW494458	homolog to myosin regulatory light chain	1.8	±	0.8
NM_207682	kinesin heavy chain member 1B	1.7	±	0.5
AK161742	kinesin light chain 1	4.3	±	1.2
NM_019410	profilin 2	6.0	±	1.3
AV148480	thymosin, beta 10	2.2	±	0.8
NM_021278	thymosin, beta 4	2.5	±	1.1
BB559082	tubulin cofactor a	2.1	±	0.5
NM_010910	neurofilament, light polypeptide	5.1	±	1.3
NM_008691	neurofilament, medium polypeptide	3.4	±	1.6
NM_010904	neurofilament, heavy polypeptide	1.9	±	0.4
**ATP biosynthesis**				
AV105788	ATPase, H+ transporting, lysosomal 34 kD, V1 subunit D	2.6	±	0.8
AV172216	ATPase, H+ transporting, lysosomal 16 kD, V0 subunit C	7.5	±	2.1
BI154058	ATPase, H+ transporting, lysosomal 13 kD, V1 subunit G	3.5	±	1.0
**sodium:potassium-exchanging ATPase activity**				
BG261955	ATPase, Na+/K+ transporting, beta 2 polypeptide	1.4	±	0.6
AV152334	ATPase, Na+K+ transporting, beta 1 polypeptide	6.2	±	0.6
**Genes implicated in ALS and neurodegeneration**				
BC002066	Superoxide dismutase 1, soluble	2.2	±	1.0
NM_007835	dynactin 1	1.5	±	0.6
NM_009496	vesicle-associated membrane protein 1	1.5	±	0.7
AI848048	amyloid beta (A4) precursor-like protein 1	3.9	±	1.3
NM_030598	Down syndrome critical region gene 1-like 1	2.9	±	0.7
NM_011170	prion protein	3.6	±	1.0
**Electron transport chain**				
**Complex I**				
NM_026610	NADH dehydrogenase (ubiquinone) 1 beta subcomplex 4	1.5	±	0.4
NM_023172	NADH dehydrogenase (ubiquinone) 1 beta subcomplex, 9	2.7	±	1.3
NM_019443	NADH dehydrogenase (ubiquinone) 1 alpha subcomplex, 1	4.2	±	0.6
NM_028177	NADH dehydrogenase (ubiquinone) 1, alpha/beta subcomplex, 1	1.8	±	0.6
NM_026614	NADH dehydrogenase (ubiquinone) 1 alpha subcomplex, 5	2.7	±	0.5
NM_025358.1	NADH dehydrogenase (ubiquinone) 1 alpha subcomplex, 9	0.7	±	0.2
NM_028388	NADH dehydrogenase (ubiquinone) flavoprotein 2	3.2	±	1.8
**Complex II**				
NM_023281	succinate dehydrogenase complex, subunit A, flavoprotein	1.5	±	0.6
**Complex III**				
NM_025407	ubiquinol-cytochrome c reductase core protein 1	0.8	±	0.6
NM_025899	ubiquinol cytochrome c reductase core protein 2	2.0	±	0.5
NM_026219	ubiquinol-cytochrome c reductase binding protein	8.0	±	1.3
**Complex IV**				
NM_009941	cytochrome c oxidase subunit IV isoform 1	1.6	±	0.3
NM_007747	cytochrome c oxidase, subunit Va	4.6	±	0.6
NM_009942	cytochrome c oxidase, subunit Vb	3.0	±	0.8
NM_025628	cytochrome c oxidase, subunit VIb polypeptide 1	5.0	±	0.5
NM_053071	cytochrome c oxidase, subunit VIc	4.3	±	1.0
NM_025567	cytochrome c-1	2.3	±	0.6
NM_007748	cytochrome c oxidase, subunit VI a, polypeptide1	5.6	±	0.8
NM_009945	cytochrome c oxidase subunit VIIa 2	5.2	±	1.0
**ATP Synthase (Complex V)**				
NM_007505	ATP synthase, H+ transporting, mitochondrial F1 complex, alpha subunit, isoform 1	1.1	±	0.7
NM_009725	ATP synthase, H+ transporting, mitochondrial F0 complex, subunit b, isoform 1	3.0	±	1.0
NM_175015	ATP synthase, H+ transporting, mitochondrial F0 complex, subunit c (subunit 9), isoform 3	3.9	±	1.2
NM_027862	ATP synthase, H+ transporting, mitochondrial F0 complex, subunit d	4.5	±	0.7
NM_007507	ATP synthase, H+ transporting, mitochondrial F1F0 complex, subunit e	1.4	±	0.1
NM_020582	ATP synthase, H+ transporting, mitochondrial F0 complex, subunit f, isoform 2	1.6	±	1.4
NM_007512	ATPase inhibitory factor 1	1.5	±	0.2
**Adenine Nucleotide Translocator**				
NM_007450	solute carrier family 25 (mitochondrial carrier, adenine nucleotide translocator), member 4	5.7	±	1.0
NM_007451	solute carrier family 25 (mitochondrial carrier, adenine nucleotide translocator), member 5	1.2	±	0.5
**oxidoreductase activity**				
AV261043	similar to superoxide dismutase	3.2	±	1.1
BG060909	stearoyl-Coenzyme A desaturase 2	6.0	±	1.3
AK009462	aldo-keto reductase family 1, member A4	4.4	±	1.5
AV018774	L-3-hydroxyacyl-Coenzyme A dehydrogenase, short chain	3.7	±	1.6
NM_010239	ferritin heavy chain	7.0	±	0.8
NM_008133	glutamate dehydrogenase	1.3	±	0.7
BB166616	peroxiredoxin 1	3.9	±	0.4
AV300942	thioredoxin-like	2.0	±	0.9
**TCA cycle**				
AV219418	lactate dehydrogenase 2, B chain	3.4	±	0.8
AV011848	malate dehydrogenase 1, NAD (soluble)	6.0	±	1.0
AV016940	malate dehydrogenase, mitochondrial	3.3	±	0.1
**Glycolysis**				
AW558862	hexokinase 1	3.3	±	1.0
BC008184	aldolase 3	4.4	±	0.4
NM_007438	aldolase 1	1.9	±	0.6
BI407347	phosphoglycerate mutase 1	1.6	±	0.5
NM_013509	enolase 2, gamma neuronal	1.2	±	0.8
**protein folding**				
NM_022310	Heat shock 70KD protein 5	1.4	±	1.0
NM_008907	peptidylprolyl isomerase A	2.9	±	0.9
BM941165	cytochrome c oxidase, subunit XVII assembly protein	1.7	±	0.3
BM210281	chaperonin subunit 6a (zeta)	1.2	±	0.8
C77287	heat shock protein 90 kDa alpha, class A member 1	4.0	±	1.3
BC006722	heat shock protein 8	1.8	±	1.3
AV038603	FK506 binding protein 1a	2.2	±	0.5
NM_009836.1	chaperonin subunit 3 (gamma)	2.1	±	0.8
NM_009964	crystallin, alpha B	2.1	±	0.6
**proteasome**				
AV263662	proteasome (prosome, macropain) 26S subunit, ATPase 2	3.4	±	1.2
AV212146	proteasome (prosome, macropain) subunit, beta type 4	2.4	±	0.4
NM_011184	proteasome (prosome, macropain) subunit, alphatype 3	2.2	±	1.3
**ubiquitin cycle**				
NM_019912	ubiquitin-conjugating enzyme E2D 2	2.9	±	1.0
BB315985	F-box and WD-40 domain protein 11	3.0	±	0.9
NM_019712	ring-box 1	3.1	±	1.4
AU080586	microtubule-associated protein 1 light chain 3 beta	5.8	±	4.2
**lysosome**				
NM_010684	lysosomal membrane glycoprotein 1	2.7	±	0.7
BB560429	lysosomal-associated protein transmembrane 4B	3.8	±	0.8
**calcium ion binding**				
NM_012061	Ca++dependent activator protein for secretion	2.1	±	0.9
BF608828	tumor protein, translationally-controlled 1 (Tpt1)	4.1	±	1.2
NM_010097	Sparc-like1	2.6	±	1.3
NM_012038	visinin-like 1	3.4	±	1.1
NM_016760	clathrin, light polypeptide	2.3	±	0.3
BG067649	epidermal growth factor receptor pathway substrate 15	2.0	±	1.1
NM_009722	ATPase, Ca++ transporting, cardiac muscle, slowtwitch 2	3.3	±	1.0
BC021347	calmodulin 2 (phosphorylase kinase, delta)	6.0	±	1.1
AV015462	calmodulin 1	2.2	±	0.6
AV103412	transketolase	3.2	±	0.7
**zinc ion binding**				
BB390675	matrin 3	4.4	±	1.0
BB833716	tetratricopeptide repeat domain	3.6	±	0.8
BC010197	Carboxypeptidase E	5.0	±	1.8
BB246182	The ring finger protein 157	3.0	±	0.9
NM_019712	ring-box 1 (Rbx1)	3.1	±	1.4
AA796766	metallothionein 2	2.1	±	0.8
NM_013602	metallothionein 1	2.6	±	0.8
**protein serine/threonine kinase activity**				
BB453775	mitogen activated protein kinase 10	3.0	±	1.4
AW541674	mitogen activated protein kinase kinase 7	2.4	±	1.4
BF456404	p21 (CDKN1A)-activated kinase 1	3.0	±	0.8
BB234940	discoidin domain receptor family, member 1	3.6	±	1.9
**Myelination**				
AV328388	myelin basic protein	11.7	±	0.2
BB768495	proteolipid protein	6.7	±	0.8
NM_008614	myelin-associated oligodendrocytic basic protein	3.5	±	1.0
NM_008885	peripheral myelin protein	3.8	±	0.7
**glutamine biosynthesis**				
AI391218	glutamine synthetase	1.9	±	0.9
**cell adhesion**				
NM_007614	catenin (cadherin associated protein), beta 1	3.0	±	1.1
BB066232	catenin (cadherin associated protein), alpha 1	2.3	±	0.7
**protein modification**				
AW551908	ubiquitin C	3.6	±	1.6
NM_011664	ubiquitin B	7.6	±	0.9
NM_019883	ubiquitin A-52 residue ribosomal protein fusion product 1	4.6	±	0.6
**vesicle-mediated transport**				
AF326545	syntaxin binding protein 1	1.8	±	0.4
**neurotransmitter secretion**				
BC018249	synaptosomal-associated protein 25 kDa	2.1	±	1.3
**nucleosome assembly**				
BB252350	H3 histone, family 3A	5.0	±	1.6
BC017540	TSPY-like 4	2.7	±	1.2
AV003424	H2A histone family, member Z	2.0	±	0.5
**cell death**				
BB433678	clusterin	4.0	±	1.9
**receptor mediated endocytosis**				
BB041005	sorting nexin 17	1.9	±	0.6
**gap junction**				
BB039269	gap junction membrane channel protein alpha 1	3.4	±	0.5
**Golgi stack**				
BE691746	serine incorporator 3	1.8	±	0.5
AB006361	prostaglandin D2 synthase (brain)	3.6	±	1.1
BC026372	Nedd4 family interacting protein 1	4.8	±	1.7
**protein transport**				
NM_012061	Ca2+ dependent activator protein for secretion	2.1	±	0.9
AF326545	syntaxin binding protein 1	1.8	±	0.4
NM_009005	RAB7, member RAS oncogene family	1.9	±	0.9
AV339290	RAB14, member RAS oncogene family	2.2	±	0.8
BB396668	RAB6B, member RAS oncogene family	2.1	±	0.5
NM_019718	ADP-ribosylation-like 3	1.8	±	0.4
BF660710	guanosine diphosphate (GDP) dissociation inhibitor 3	2.3	±	0.8
**nuclear mRNA splicing, via spliceosome**				
BC003745	DEADH (Asp-Glu-Ala-AspHis) box polypeptide 15	1.9	±	0.7
**positive regulation of transcription**				
BB114677	DEAD (aspartate-glutamate-alanine-aspartate) box polypeptide 5	2.0	±	0.7
NM_007614	catenin beta	3.0	±	1.1
**protein targeting**				
AV209126	signal recognition particle 14 kDa (homologous Alu RNA binding protein)	3.9	±	0.8
BB100384	tyrosine 3-monooxygenasetryptophan 5-monooxygenase activation protein, zeta	2.1	±	0.6
AV124281	tyrosine 3-monooxygenasetryptophan 5-monooxygenase activation protein, theta	4.3	±	1.2
NM_018871	3-monooxgenasetryptophan 5-monooxgenase activation protein, gamma polypeptide	2.9	±	0.8
BB106523	tyrosine 3-monooxygenasetryptophan 5-monooxygenase activation protein, epsilon	1.9	±	0.9
AV021552	tyrosine 3-monooxygenasetryptophan 5-monooxygenase activation protein, beta	5.4	±	0.3
**Transcription**				
BB357514	TSC22 domain family, member 1	5.1	±	0.3
AF201285	TSC22-related inducible leucine zipper 1b	2.4	±	0.4
BB462744	retinoblastoma binding protein 4	3.3	±	1.5
AV122231	MORF-related gene X	3.7	±	1.2
AJ414734	53BP1 protein	2.8	±	1.3
**regulation of progression through cell cycle**				
BC003856	Protein phosphatase 2, catalytic subunit, α isoform	1.8	±	0.6
AW907805	RAN, member RAS oncogene family	2.4	±	0.5
BB531646	Protein phosphatase 2, regulatory subunit, α isoform	6.4	±	3.8
**Miscellaneous**				
NM_133354	SMT3 (supressor of mif two, 3) homolog 2	4.4	±	0.7
NM_007694	chromogranin B	1.5	±	0.7
BC002008	fatty acid binding protein 5, epidermal	5.5	±	1.1
NM_030692	SAC1 (supressor of actin mutations 1)	1.7	±	0.6
NM_021881	quaking protein	2.4	±	1.1
NM_008907	peptidylprolyl isomerase A	2.9	±	0.9
NM_053076	reticulon 3	4.9	±	1.3
AV028400	poly(A) binding protein, nuclear 1	2.8	±	1.0
AK016567	glycoprotein m6b	2.0	±	1.0
BB381966	integrator complex subunit 6	4.3	±	1.0
NM_008248	histidine triad nucleotide binding protein 1	6.3	±	0.9
BB005298	prolyl endopeptidase	3.1	±	1.5
AV006122	N-myc downstream regulated gene 4	6.7	±	0.8
NM_024166	coiled-coil-helix-coiled-coil-helix domain containing 2	8.9	±	1.1
NM_145942	3-hydroxy-3-methylglutaryl-Coenzyme A synthase 1	6.1	±	0.4
AW742814	methionine aminopeptidase 2	1.7	±	0.5
BB274009	protein kinase, cAMP dependent regulatory, type I beta	3.3	±	1.2
AV148646	acetyl-Coenzyme A acetyltransferase 2	3.0	±	1.5
BI438046	ornithine decarboxylase antizyme	4.3	±	0.7
AV172168	calponin 3, acidic	2.2	±	0.6
AV348702	inositol (myo)-1(or 4)-monophosphatase 1	1.9	±	0.3
BB168483	chloride channel, nucleotide-sensitive, 1A	2.3	±	0.4
AA038464	Nur77 downstream gene 2	1.8	±	0.3
BB251922	cyclic nucleotide phosphodiesterase 1	6.4	±	0.3
AV207950	phosphatidylethanolamine binding protein 1	5.0	±	1.1
BB043450	protein tyrosine phosphatase 4a1	4.9	±	1.7
AV296285	beclin 1 (coiled-coil, myosin-like BCL2-interacting protein)	2.9	±	0.3
NM_010490	CCR4 carbon catabolite repression 4-like	2.4	±	1.1
NM_007756	complexin 1	6.2	±	2.3
BC001991	selenoprotein P, plasma, 1	2.9	±	0.6
BG967663	creatine kinase, brain	1.9	±	0.5
AI987693	gag protein	4.5	±	1.6
AV124445	necdin	4.0	±	1.1
AV019984	diazepam binding inhibitor	2.7	±	1.1
AV309418	N-myc downstream regulated 1	2.8	±	1.0
AV310432	retinoblastoma binding protein 7	2.5	±	0.8
BB722680	heterogeneous nuclear ribonucleoprotein K	4.9	±	0.9
BM200248	paternally expressed 3	3.0	±	1.2

* intensity index, the average of signal intensities on three arrays divided by 1,000; SD, standard deviations among three arrays.

These array results indicated that some mRNA species were more susceptible to oxidative damage, which is consistent with our previous observations in Alzheimer's brains [12] and cultured primary neurons under oxidative stress [13]. Thus, RNA oxidation is not random, but highly selective. The highly abundant mRNA species, β-actin and MAP2 have only very low level of mRNA oxidation, indicating selective mRNA oxidation was not due to the abundance of mRNA species. We also examined whether selective mRNA oxidation was due to the up-regulation of mRNA expression. Quantitative RT-PCR analysis revealed that those highly oxidized mRNA species, such as ribosome protein S6, cytochrome c oxidase Va, cytochrome c and MBP mRNAs, were not up-regulated in SOD1^G93A^ spinal cords compared to non-transgenic spinal cords (Fig. 4B). However, the up-regulation could exist within specific cell types, e.g. motor neurons.

The above described 15A3 immunofluorescent staining (Fig 2) indicated that at pre-symptomatic stage, increased RNA oxidation primarily occurred in motor neurons and oligodendrocytes, not in astrocytes and microglia. The array results showed that glial cell specific mRNA species such as excitatory amino acid transporter 2 (EAAT2) and glial fibrillary acid protein (GFAP) mRNAs, which are very abundant in the glial cells, were not present in the oxidized mRNA pool. This indicates the specificity of the identified oxidized mRNA species.

### Some proteins corresponding to oxidized mRNA species are decreased

**Figure 5:**
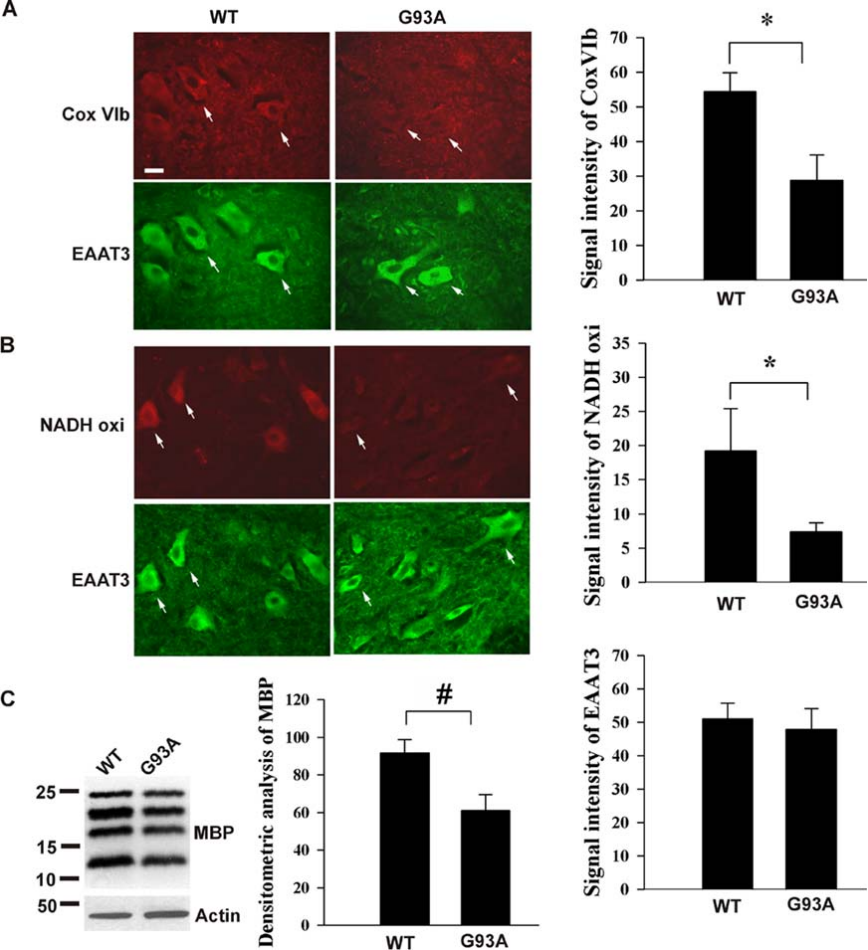
Some proteins corresponding to oxidized mRNA species are decreased. (A & B) immunofluorescent staining of lumbar spinal cord sections prepared from indicated mice (n = 3) showed that downregulation of protein levels in SOD1^G93A^ mice was found in cytochrome c oxidase VIb (*Cox VIb*) and NADH-ubiquinol oxidoreductase subunit 39 kDa (*NADH oxi*), whose mRNAs were highly oxidized but not EAAT3 protein, whose mRNA was not oxidized. Statistic analysis of immunoreactivity within motor neurons (n = 20) is shown. *P<0.0001 (C) Immunoblot analysis showed that MBP, whose mRNA was oxidized, was decreased. Densitometry analysis (standardized by actin intensity) of immunoblot is shown (n = 3). #*P*<0.01.

Our previous studies demonstrated that oxidative modification of mRNA causes reduced protein expression [11], [12]. We examined protein expression levels for the oxidized mRNA species. Since the oxidized mRNAs primarily occur in motor neurons, which are small portion of total spinal cord cell population, immunoblot analysis was not sensitive enough to detect the change, so immunofluorescent staining was performed. The results showed that some proteins, whose mRNAs were highly oxidized, were significantly decreased in the motor neurons of 60 day-old SOD1^G93A^ lumber spinal cords, such as cytochrome c oxidase VIb (Fig. 5A, *Cox VIb*) and NADH-ubiquinol oxidoreductase subunit 39 kDa (Fig. 5B, *NADH oxi*). On the other hand, the neuronal glutamate transporter EAAT3, whose mRNA was not oxidized, was not decreased. Further, the white matter oligodendrocytes also showed significant mRNA oxidation in SOD1^G93A^ mice (Fig. 2). MBP, whose mRNA was oxidized, was decreased as determined by immunoblotting (Fig. 5C). These results suggested that some proteins corresponding to oxidized mRNA species may be decreased.

### Vitamin E reduces mRNA oxidation in SOD1^G93A^ mice

**Figure 6:**
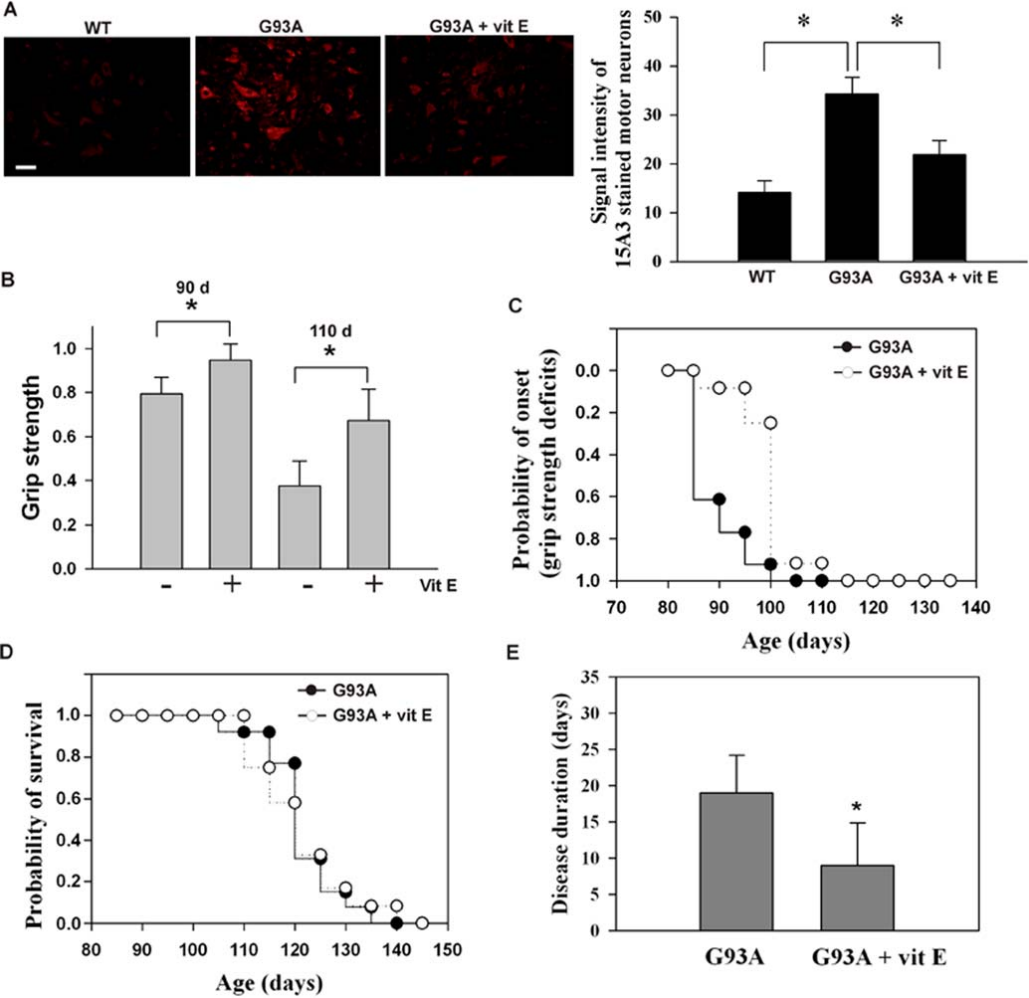
Vitamin E reduces RNA oxidation and delays the disease onset, but not the survival. (A) immunofluorescent staining of lumbar spinal cords sections prepared from indicated mice showed that RNA oxidation is significantly decreased in vitamin E treated mice (n = 3 per group). Statistical analysis of immunoreactivity within motor neurons (n = 20) is shown. *P<0.01 (B & C) The decline in motor performance by measuring grip strength was significantly delayed (∼14 days, *P<0.005) in the vitamin E treated mice. There was no significant difference in the life span between the vitamin E treated and non-treated mice (D). The disease duration is significantly shortened (*P<0.01) (E). n = 17 each group.

We investigated whether mRNA oxidation contributes to motor neuron degeneration. We first tested if vitamin E would reduce mRNA oxidation. SOD1^G93A^ mice were orally fed with vitamin E (200 IU/day per mouse, 5 days/week) at the age of 30-days (n = 3 per group). The dosage of vitamin E was chosen based on high dose vitamin E therapy in ALS patients [24]. After 30 days of treatment (at age of 60-day), lumbar spinal cords were harvested for analysis. Immunofluorescent staining (Fig. 6A) and immunoprecipitation analysis (Fig 4A, compared *G93A* with *G93A+vitE* in oxidized mRNA pool) revealed that mRNA oxidation level was significantly decreased in the treated mice. The protein expression levels for cytochrome c oxidase VIb, NADH-ubiquinol oxidoreductase subunit 39 kDa and myelin basic protein were partially restored in the treated mice when compared to the non-treated mice (data not shown). These results indicated that vitamin E treatment can reduce mRNA oxidation in SOD1^G93A^ mice.

### Reduced mRNA oxidation by vitamin E delays disease onset, and improves motor performance, but does not change survival

mRNA oxidative damage occurs at an early age of mice (Fig. 2), but significant protein, lipid and DNA oxidative damage does not occur until active disease progression stage [25]–[27]. Thus, any protective effects by vitamin E in the early symptomatic stage may be primarily due to reduced mRNA oxidation. We next investigated if the reduction of mRNA oxidation by vitamin E would delay the progression of ALS. The SOD1^G93A^ mice were orally fed with vitamin E starting at the age of 30-days until they reached the end stage of disease. Motor performance was monitored by measuring grip strength. We found there was a 23% decline in grip strength in the non-treated mice (n = 17) but only an 8% decline in the treated mice at 90 days (n = 17); there was a 60% decline in the non-treated mice and a 40% decline in the treated mice at 110 days (Fig. 6B). The cumulative probability of onset, as defined by the grip strength deficit, was significantly delayed (by ∼15 days, *p*<0.01) in the treated mice (106±6.1 days), compared to the non-treated mice (91±4.3 days) (Fig. 6C). However, the mean life span was not significantly different between the treated mice (123±7.6 days, n = 12) and the non-treated mice (122±7.3 days, n = 13) (Fig. 6D). The disease duration, defined as the period from the time of 50% decline in grip strength to the time of death, was significantly shortened in the treated mice (10±4.8 days), compared to the non-treated mice (17±6.6 days, *p*<0.01) (Fig. 6E). These results indicated that decreased mRNA oxidation by vitamin E can delay disease onset, which is consistent with the previous study by Gurney *et al.* [28].

### Reduced mRNA oxidation by vitamin E delays the progressive loss of motor neurons, ubiquitin aggregation, and gliosis, and significantly reduces mitochondria vacuolization in motor neurons

**Figure 7:**
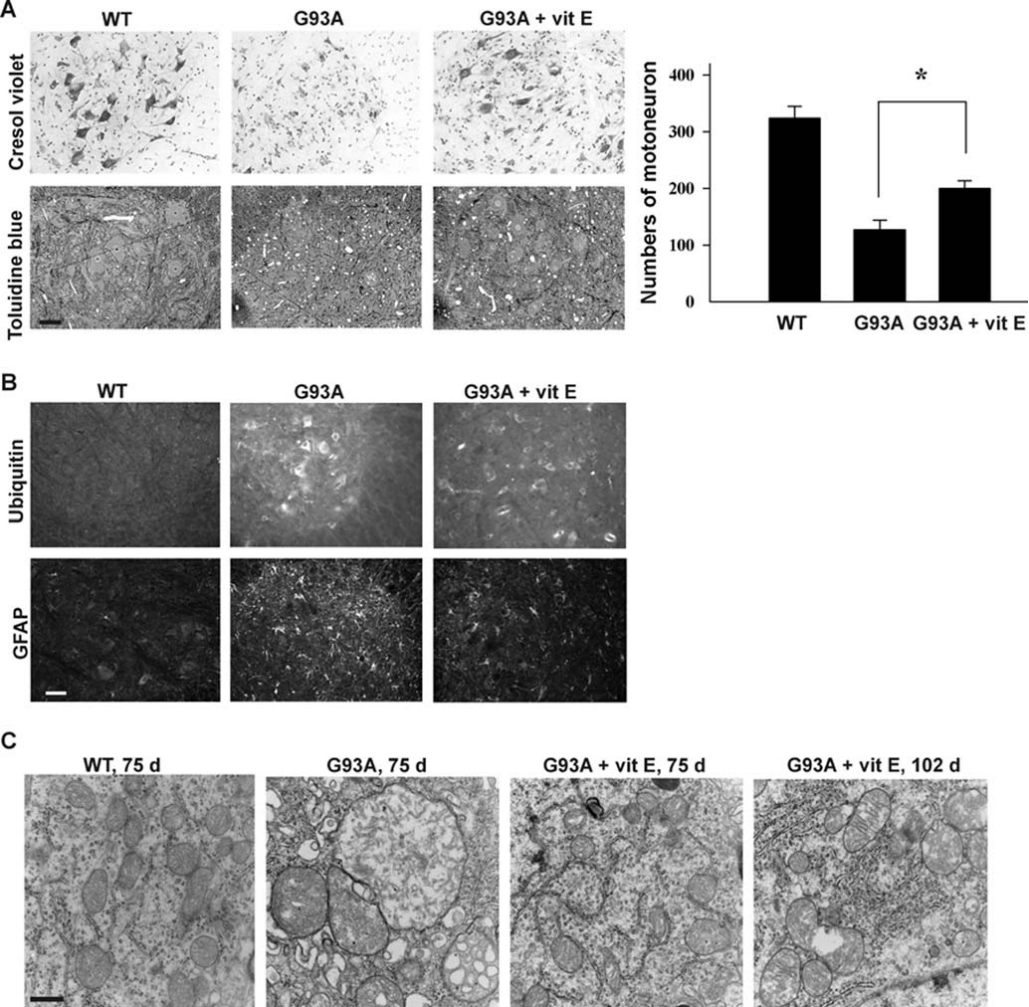
Effect of vitamin E on the course of disease in SOD1G93A mice. (A) Cresyl violet-stained and toluidine blue-stained sections through the ventral horn of lumbar spinal cord showed partial preservation of neurons in vitamin E treated mice compared with non-treated mice at age of 100 days. The number of motor neurons in the lumbar spinal cord was counted (n = 3 per group, *P<0.01). Vitamin E delays the progressive loss of motor neurons. (B) Immunofluorescent staining of lumbar spinal cord sections showed that gliosis (GFAP staining) and ubiquitin aggregation (ubiquitin staining) were significantly reduced in vitamin E treated mice (100 day-old). The ventral horn area is shown. Scale bar, 10 µm. (C) Electron microscopy shows that vitamin E significantly reduces mitochondria vacuolization in motor neurons. Scale bar, 0.5 µm.

One striking observation in the above onset study (Fig. 6) was that at the age of 100 days, the non-treated mice exhibited grip strength deficit and were sick while the treated mice still had normal motor performance and remained active. However, between 110 and 120 days of age, the treated mice developed onset rapidly and died within a similar age range as the non-treated mice. We therefore decided to examine the pathological changes at 100 days and 120 days of age. To examine the numbers and morphology of the motor neurons, we performed cresol violet staining on the lumbar spinal cord sections from non-treated and treated mice (n = 3 per group). The results showed that at 100 days of age, numerous of motor neurons were lost in the non-treated mice, and the remaining motor neurons were atrophic. In contrast, there was less motor neuron loss in sections from the treated mice, and the motor neurons were healthier in appearance, compared to the non-treated mice (Fig. 7A). However, at 120 days of age, there was no obvious difference between treated and non-treated mice samples, in that the majority of motor neurons had degenerated (not shown). We also performed toluidine blue staining (Fig. 7B), and the results were consistent with the cresol violet staining results. Importantly, there was significant vacuolization in the non-treated mice and only a slight vacuolization in the treated mice at 100 days of age. These results clearly indicated that vitamin E can reduce mRNA oxidation, restore protein expression level and partially protect motor neurons up to 100 days of age. Notably, significant protein oxidation [25], lipid peroxidation [26] and DNA oxidation [27] occurs at active disease progression stage; thus, the observed protective effects by vitamin E are primarily due to reduction of RNA oxidation.

We examined the effects of vitamin E on the occurrence of the following events associated with SOD1^G93A^ mice: gliosis, ubiquitin aggregation, and mitochondria vacuolization. At 100 days of age, both GFAP immunostaining, a marker for reactive gliosis, and ubiquitin immunostaining, a marker for protein aggregation, were significantly reduced in the treated mice, compared to the non-treated mice (Fig. 7B). However, at 120 days of age, no obvious difference was observed (not shown), suggesting that these are probably secondary events to motor neuron degeneration. Further, we performed electron microscopy to examine mitochondrial morphology of motor neurons. As shown in Figure 7C, severely swollen mitochondria with disorganized and dilated cristae were observed in the motor neurons of 75 day-old SOD1^G93A^ mice. Significantly, the treated mice exhibited mostly normal mitochondrial morphology at 75 days, and even at 102 days, the mitochondria only slightly vacuolated, and the cristae were clear (not severely damaged). These results suggest that mitochondria vacuolization may be highly associated with RNA oxidation. One important result supporting this possibility is that, as described above, many mRNAs encoding proteins involved in mitochondrial functions were highly oxidized (Table 1).

## Discussion

In the present study, we found that mRNA oxidation is a common feature in ALS patients and mutant SOD1 transgenic mice and also an early event preceding motor neuron degeneration. This study suggests that mRNA oxidation may contribute to motor neuron deterioration in ALS.

We used 15A3 antibodies to separate oxidized mRNAs from non-oxidized mRNAs and subsequently quantified and identified oxidized mRNAs. The specificity of 15A3 antibody is very important. This was validated by including the no antibody control and the 8-OHG-blocked antibody control in each experiment. In addition, we analyzed the 15A3-precipitated mRNAs by HPLC-ECD (high-performance liquid chromatography coupled with electrochemical detection) and confirmed that the mRNAs recognized by 15A3 contained high levels of 8-OHG. This indicates that the isolated/detected mRNAs were oxidized mRNAs.

In the ALS postmortem tissues study (Fig. 1), we found that mRNAs are oxidatively damaged to a variable extent in ALS patients. Interpretation of human postmortem tissue pathological changes is difficult, since postmortem tissue represents the very end stage of the neurological disease and reflects the cells that remain but not necessarily those that are at risk. The mutant SOD1 mice studies provide the valuable information of the role of RNA oxidation in ALS. From the results of the SOD1^G93A^ mice study, we speculate that significantly increased mRNA oxidation may occur in the motor neurons and oligodendrocytes of ALS-affected areas at the prodromal stage. This may contribute to neuronal deterioration and in combination with other toxicities eventually leading to motor neuron degeneration.

Previous studies in SOD1^G93A^ mice showed that significant increases in lipid, protein and DNA oxidation occurs during symptomatic stage [25]–[27]. We found that mRNA oxidation occurs as early as 45 days, progressively increases with age, until it peaks at 60–70 days of age, and then diminishes when the motor neurons begin to degenerate (Fig. 2). Those motor neurons showing RNA oxidation appear to be still healthy as judged by the nuclear and chromatin morphology and mitochondrial morphology (Fig. 3). These results indicate that mRNA oxidation is an early event.

RNA oxidation primarily occurs in motor neurons and oligodendrocytes at pre-symptomatic stage (Fig. 2). This indicates that both cell types are more vulnerable to RNA oxidation. Like neurons, oligodendrocytes are highly vulnerable to injury by oxidative stress [29]. Oligodendrocytes, compared to other cells, have a high lipid content, high iron content, and low supplies of cellular antioxidant [30].

Increased mRNA oxidation also occurs in the pre-symptomatic stage of mice expressing other mutant SOD1 (Fig. 1E). Mutant SOD1^G37R^, like SOD1^G93A^, retains full dismutase activity [31]. Both SOD1^G85R^ and SOD1^G127X^ lack dismutase activity and are unstable [32], [33]. Mutant SOD1 ^H46R/H48Q^ possesses little or no SOD1 activity [34]. The protein levels of mutant SOD1 in these mice are different: SOD1^G93A^, SOD1^G37R^, SOD1^G85R^ and SOD1^G127X^ mice express 17-, 5-, 0.9- and 0.45-fold human mutant SOD1 of mouse wild type SOD1, respectively [20]. This suggests that increased RNA oxidation is a common feature in these ALS mouse models and has nothing to do with SOD1 activity or mutant SOD1 expression level. What could be the possible mechanism underlying ROS formation in these mutant SOD1 mice? We would like to propose one possible mechanism. Ferri *et al.* have demonstrated that one common property of different FALS-mutant SOD1s with widely differing biophysical properties is the association with mitochondria to a much greater extent than wild-type SOD1 [35]. Mutant SOD1 proteins associated with the mitochondria tend to form cross-linked oligomers and impair mitochondrial function, which could lead to increased ROS formation.

Identification of oxidized mRNA species by DNA microarray revealed that some mRNA species are more susceptible to oxidative damage, which was also observed in our previous studies [11], [12]. The phenomenon of selective RNA oxidation was not related to the abundance of mRNA species or the up-regulation of mRNA expression. No common motifs, sequences or structures were found in oxidized mRNA species at this time. Several possible mechanisms are currently under investigation.

A very striking finding in this study is that many identified known oxidized mRNAs are related to ALS: (1) mRNAs corresponding to genes linked to familial ALS or ALS-like human motor neuron disease, including SOD1, dynactin 1, and vesicle-associated membrane protein 1 (VAMP) mRNAs. A missense mutation in the p150 subunit of the *dynactin* (*DCTN1*) gene has been described in a human kindred with a slowly progressive, autosomal dominant form of lower motor neuron disease [36], [37]. A dominant missense mutation in the *VAMP-B* gene (ALS8) has been linked to an atypical ALS that is accompanied by an unusual tremor [38]. VAMP-B interacts with VAMP-A involving in vesicular trafficking [39]. (2) mRNAs encoding neurofilament subunits. The genes encoding three neurofilament subunits have long been suspected as causative for ALS because of their link with motor neuron pathology in mice and humans [40]. (3) mRNAs encoding proteins involved in protein folding and degradation. Reduction of proteasome 26S function and protein chaperone activities have been found in SOD1^G93A^ transgenic mice [41], [42]. Impaired function of protein folding and degradation pathway can lead to protein aggregation, one of hallmarks of ALS. (4) mRNAs encoding proteins involved in the mitochondrial electron transport chain (ETC). Dysfunction of mitochondrial ETC has been previously found in SOD1^G93A^ mice and ALS patients [43], [44]. It is possible that mRNA oxidation is responsible for the observed dysfunction and also the mitochondria vacuolization (Fig. 7C). (5) mRNAs encoding proteins involved in glycolysis and tricarboxylic acid (TCA) cycle. Depleted ATP levels and reduction of glucose use have been reported in spinal cords of SOD1^G93A^ mice at pre-symptomatic stage [45]. Abnormal glycolysis and TCA process combined with mitochondrial electron transport chain dysfunction could result in ATP synthesis impairment. (6) mRNAs encoding metallothioneins. Metallothioneins, known to bind copper ions and decrease oxidative toxicity, have been suggested to have important roles in the pathophysiology of ALS [46]–[48]. (7) mRNAs encoding proteins involved in protein transport. Defective axonal transport has been found to cause late-onset progressive degeneration in transgenic mice [49]. Dominant point mutations in dynein causing motor neuron disorders have been found in both ALS patients and mouse models [37]. (8) mRNAs encoding proteins involved as structural constituents of myelin sheath, including MBP, proteolipid protein (PLP) and myelin-associated oligodendrocytic basic protein (MOBP). MBP was reported to be related to axon degeneration [50]. MBP protein level is significantly decreased in ALS spinal cords [51]. We show that MBP protein is significantly decreased in the presymptomatic stage of SOD1^G93A^ spinal cords (Fig. 5C). mRNA oxidation may be responsible for loss of MBP protein. Myelin sheaths contribute to the structure and stability of the axons [52]. Abnormally expressed myelin proteins may affect axon stability and contribute to axon degeneration.

We examined protein expression levels for the oxidized mRNA species and found that some proteins corresponding to oxidized mRNA species are decreased (Fig. 5). Our previous studies demonstrated that oxidized bases in mRNAs can cause ribosome stalling on the transcripts, leading to decreased protein expression [13]. A recent study [14] demonstrates that oxidized mRNA induces translation errors, producing short polypeptides because of premature termination or translation error-induced degradation.

Does mRNA oxidation contribute to the pathogenesis of the disease? We approached this question by treating SOD1^G93A^ mice with vitamin E. As mentioned above, RNA oxidation occurs at an early age of mice, but significant protein, lipid and DNA oxidations do not occur until active disease progression stage [25]–[27]; thus, the protective effects by vitamin E in the early symptomatic stage may be primarily due to reduced mRNA oxidation. Four observations strongly support that mRNA oxidation does contribute to the disease. First, the protein expression levels for the oxidized mRNA species are decreased (Fig. 5). Second, the vitamin E treated mice still had normal motor performance and were still active at the age of 100 days while the non-treated mice were already sick (Fig. 6). The treated mice developed onset rapidly between 110 and 120 days and died within a similar age range as the non-treated mice. This is probably because reduced mRNA oxidation by vitamin E partially diminishes toxicity in motor neurons, but toxicities from other non-neuronal cells are still present. Third, reduced mRNA oxidation by vitamin E significantly reduces mitochondria vacuolization in motor neurons (Fig. 7C). Finally, many identified known oxidized transcripts are related to ALS. RNA oxidation may account for many neuropathological changes reported previously.

Dietary supplement with vitamin E is widely used in the clinical as an antioxidant for ALS patients. Although in previous studies vitamin E did not prolong survival of ALS patients [24], Ascherio *et al.* found that those individuals who took vitamin E supplements for 10 years had less than 50% risk of death from ALS than that of vitamin E nonusers [53]. These studies support our observations in SOD1^G93A^ mice that RNA oxidation is an early event and contributes to motor neuron degeneration. Thus, blocking RNA oxidation at prodromal stage may prevent/slow the disease progression; however, at the disease stage, the antioxidant treatment may be already too late so there is no significant beneficial effect.

## Methods

### ALS tissues

ALS brain and spinal cord tissues were obtained from the Johns Hopkins ALS Brain Bank. Postmortem delays for autopsy were 7.3±0.8 hr. Pathological confirmation of ALS was made on all specimens by standard histological evalution of spinal cord and motor cortex, with use of hematoxylin and eosin to evaluate motor neuron loss, and with myelin stains (Luxol-fast blue) to establish corticospinal tract degeneration.

### Animal model

All animal experiments were performed at Ohio State University under national and institutional guidelines using protocols approved by the the Animal Care & Use Committee of Ohio State University. Human SOD1^G93A^ transgenic mice (B6SJL-Tg [SOD1-G93A] 1Gur with high copy number of the mutant human SOD1 gene) and human wild-type SOD1 transgenic mice (B6SJL-Tg [SOD1-G93A] 2Gur) were purchased from Jackson Laboratory (Bar Harbor, ME). Adult same gender mice were housed 5 per cage, under 12 h dark/light cycles. Transgene was determined by PCR using genomic DNA extracted from tail biopsies. From the time when transgenic mice showed motor deficits, food and water were placed on the cage floor. Transgenic animals were killed when they can not right themselves within 30 sec.

### Tissue collection and RNA isolation

After decapitation, spinal cords were removed and dissected rapidly. The samples were placed in individual tubes and homogenized in 1 ml of Trizol reagent (Invitrogen, Carlsbad, CA). RNA isolation was performed according to the manufacturer's protocol. After DNase I (Invitrogen) treatment, poly(A)^+^ RNAs were isolated using the Oligo-tex mRNA Purification Kit (Qiagen Inc., Valencia, CA).

### Immunohistochemistry

Mice were perfused transcardially with 4% paraformaldehyde after being deeply anesthetized with tribromoethanol (Avertin; 200 µl/10 g intraperitoneally). The brains and spinal cords were rapidly removed and cryoprotected with 20% sucrose for 24 h. The sections were blocked in 8% normal goat serum (NGS; Vector Laboratories, Burlingame, CA) and 0.1% Triton X-100 in Tris-buffered saline (TBS) for 60 min and incubated in primary antibody solution overnight at 4°C. After thorough washing with TBS, the sections were then incubated with secondary antibody in TBS containing 2% NGS for 60 min at 4°C followed by thorough washing with TBS. The following primary antibodies were used in this study: rabbit anti-ubiquitin pAb (1∶50, Santa Cruz Biotechnology, Inc., Santa Cruz, CA ), mouse anti-8-OHG mAb (1∶250, QED Bioscience, San Diego, CA), rabbit anti-EAAT3 pAb (1∶1000), mouse anti-NADH-ubiquinol oxidoreductase 39 kDa Subunit, (1∶2000, Molecular Probes, Carlsbad, CA), mouse anti-cytochrome c oxidase VIb Subunit (1∶750, Molecular Probes). Images were obtained using a Zeiss Axioskop 2 upright microscope and AxioVision software.

### Tissue preparation and Electron microscopy

Mouse spinal cords were initially fixed by intracardiac perfusion with a solution of 0.1% sodium phosphate (pH 7.6), 4% paraformaldehyde, and 2.5% glutaraldehyde, and postfixed in 1% osmium tetroxide, and embedded in Eponate 12 (Ted Pella, Redding, CA). One-micrometer sections were stained with toluidine blue. Motor neurons were identified by their large size (>20 µm) and distinct nuclear morphologies in the ventral horn of the lumbar cord.

### Cresyl violet staining and motor neuron counts

For motor neuron counting, the L5 lumbar spinal cords were sectioned at 20 µm with a Microm cryostat at −25°C. Every fifth section was stained with 0.1% (w/v) cresyl violet solution. Motor neurons in the ventral horn were quantified by counting large pyramidal neurons that stain with cresyl violet and possess a prominent nucleolus. 14 sections of spinal cord were counted per mouse, and the analysis was performed blindly. Three animals per genotype were analyzed.

### Immunoprecipitation

Poly(A)^+^ RNAs (1.5 µg) were incubated with 1.5 µg of anti-8-OHG antibody (15A3) at room temperature for 4 hr. For negative controls, the primary antibody was omitted or pre-incubated with 24 ng/µl of 8-OHG (Cayman Chemical, Ann Arbor, MI). Immobilized Protein L gel beads (20 µl) (Pierce, Rockford, IL) were added and incubated at 4°C for an additional 15 hr. The beads were washed three times with PBS and 0.04% (v/v) NP-40 (Roche Applied Sciences, Indianapolis, IN). Afterward, the following items were added in the following order: 300 µl of PBS with 0.04% NP-40, 30 µl of 10% (w/v) SDS, and 300 µl of PCI (phenol∶chloroform∶isoamyl alcohol, 25∶24∶1). The mixture was incubated at 37°C for 15 min (vortex every 5 min) and separated to aqueous phase and organic phase by spinning at 14,000 rpm for 5 min. The aqueous layer was collected and mixed with 40 µl of 3 M sodium acetate, pH 5.2, 2 µl of 5 mg/ml glycogen, plus 1 ml of 95% (v/v) ethanol. The sample was frozen at −80°C for 1 hr and centrifuged for 20 min. The pellet was washed with 75% ethanol, air-dried and then resuspended in 14 µl of DEPC-treated H_2_O.

### cDNA synthesis and Southern blotting

Immunoprecipitated mRNAs were reversely transcribed using avian myeloblastosis virus (AMV) reverse transcriptase kit (Roche Applied Sciences). For 15 µl of reaction mixture, 7 µl of immunoprecipitate mRNAs and 1 µl (0.75 µg) of oligo-(dT) _24_-T7 primer were mixed and incubated at 70°C for 10 min. After 2 min on ice, the master mix contained 7 µl of 5× first-strand buffer, 0.5 mM 2′-deoxynucleoside 5′-triphosphates [deoxy (d)-ATP, dCTP and dGTP], 0.13 mM 2′-deoxythymidine-5′-triphosphate, 0.03 mM digoxigenin-11-2′-deoxy-uridine-5′-triphosphate (Roche Applied Sciences), 2.5 U of RNase Inhibitor (Invitrogen), and 10 U of AMV reverse transcriptase. The mixture was incubated at 42°C for 90 min. cDNAs were resolved in 1% agarose gel and then transferred electrophoretically to a positively charged nylon membrane (Roche Applied Sciences) using the Trans-Blot SD semidry transfer system (Bio-Rad, Hercules, CA) according to the directions of the manufacturer. Digoxogenin labeled on cDNAs was detected with a Digoxogenin High Prime DNA Labeling and Detection Starter Kit II (Roche Applied Sciences).

### Microarray hybridization

Preparation of cRNA was performed according to the Two-cycle cRNA amplification protocol provided by Affymetrix (Santa Clara, CA). The immunoprecipitated oxidized mRNA was converted to cDNA using AMV reverse transcriptase and a GeneChip T7-oligo(dT) primer 5′-GGCCAGTGAATTGTAATACGACTCACTATAGGGAGGCGG-(dT)_24_-3′(Affymetrix). For the first-cycle IVT amplification of cRNA, a MEGAscript T7 kit (Ambion Inc., Austin, TX) was used. The cRNA was purified and cleanup by using RNeasy mini kit (Qiagen). Each cRNA sample was synthesized from 2 independent biological samples. Biotin-labeled cRNA was synthesized from second-cycle cDNA using an IVT Labeling Kit (Affymetrix). The yield of the *in vitro* transcription reaction was determined by product absorbance at 260 nm measured by NanoDrop ND-1000 (NanoDrop Technologies, Inc., Montchanin, DE). Size of cRNA probes was evaluated by using RNA 6000 Nano LabChip Kit (Agilent, Palo Alto, CA, USA). Experion Automated Electrophoresis System was used to examine the quality of samples (Affymetrix). Fragmented cRNA (15 µg) was used for hybridization to GeneChip Mouse Genome 430 2.0 arrays (Affymetrix).

### RT-PCR

Immunoprecipitated mRNAs were reversely transcribed with AMV reverse transcriptase (Roche) and Oligo-(dT)_24_ primer. PCRs were performed in the presence of 3 mM MgCl_2_, 0.2 mM dNTP, 0.25 µM primers and 2 U Taq DNA polymerase (Invitrogen) in 1× PCR buffer, with gene–specific primers, including Cu/Zn SOD1 F (5′-atggcgacgaaggccgtgtgcgt) and R (5′-ctggcaaaatacaggtcattgaaacagaca), MAP2 F (5′-ttggctcacttgacaatgctcacc) and R (5′-aatatgacacctgctcagagccca-3′), MBP F (5′-agcatgccttctgtagaccttcca) and R (5′-agtaggtgcttctgtccagccata), ribosomal protein S_6_ F (5′-tgatgtccgccagtatgttgtcag) and R (5′-tggcttccttcattcttggc), Cox Va F (5′-gacattgatgcctgggaattgcgt) and R (5′-ttacactttgtcaaggcccagctc), Cyc C F (5′-tgttcactggcctctttcaggtca) and R (5′-ccagttatgtgtgtttctgagtggg), PCM F (5′-aaagaacctgaaacagtgggagcc) and R (5′-ccaaatgtcacaatgaagggtggg). A series of cycles (25, 30, 35 cycles) was performed (95°C for 30 s, specific annealing temperature to each set of primers for 45 s, and 72°C for 1 min). PCR products were visualized as single bands on 1% agarose gels stained with ethidium bromide.

### Immunoblotting

Immunoblotting was performed as described previously [54]. Briefly, protein extracts were generated from spinal cord, resolved by SDS-PAGE and transferred onto PVDF membranes. The following primary antibodies were used: rabbit anti-MBP pAb (1∶3000; Chemicon International, Inc, Temecula, CA), goat anti-β-Actin (1∶3000; Santa Cruz, CA). The immunoreactive bands were detected using the SuperSignal West Pico Chemiluminescent Substrate (Pierce Biotechnology, Rockford, IL) according to manufacturer's directions.

### Vitamin E treatment

SOD1^G93A^ transgenic (n = 32) mice were treated with vitamin E, and their littermates SOD1^G93A^ (n = 32) and wild type mice (n = 32) were used as controls. Mice were orally fed with vitamin E (200IU/each for 5 day/week) from daily supplemental softgels (Meijer Distribution, INC., Grand Rapids, MI), and started at age of 30-days. The same gender littermates of wild type, G93A, and vitamin E-treated G93A transgenic mice were used to compare their onset, survival, and pathological changes, although no significant variation of onset and survival were detected between different genders.

### Grip Strength

An objective assessment of neuromuscular performance was obtained by measuring peak force generated using a Digital Grip Strength Meter outfitted with a Hind Limb Pull Bar Assembly (Columbus Instruments). Five repetitions were taken and the average was determined for each mouse. The examiner was unaware of the genotypes of the mice during measurement. Once each litter of mice reached the age of 70 days, the grip strength was measured twice a week, on the same day of each week and at the same time of each scheduled day (within 2 h).

### Statistical analysis

The quantitative data in this study were expressed as the mean±SEM. Statistical analysis was performed using the unpaired Student's t-test.

## References

[1] Barber SC, Mead RJ, Shaw PJ (2006). Oxidative stress in ALS: a mechanism of neurodegeneration and a therapeutic target.. Biochim Biophys Acta.

[2] Fiala ES, Conaway CC, Mathis JE (1989). Oxidative DNA and RNA damage in the livers of Sprague-Dawley rats treated with the hepatocarcinogen 2-nitropropane.. Cancer Res.

[3] Wamer WG, Yin JJ, Wei RR (1997). Oxidative damage to nucleic acids photosensitized by titanium dioxide.. Free Radic Biol Med.

[4] Nunomura A, Perry G, Pappolla MA, Wade R, Hirai K (1999). RNA oxidation is a prominent feature of vulnerable neurons in Alzheimer's disease.. J Neurosci.

[5] Nunomura A, Perry G, Hirai K, Aliev G, Takeda A (1999). Neuronal RNA oxidation in Alzheimer's disease and Down's syndrome.. Ann N Y Acad Sci.

[6] Zhang J, Perry G, Smith MA, Robertson D, Olson SJ (1999). Parkinson's disease is associated with oxidative damage to cytoplasmic DNA and RNA in substantia nigra neurons.. Am J Pathol.

[7] Guentchev M, Siedlak SL, Jarius C, Tagliavini F, Castellani RJ (2002). Oxidative damage to nucleic acids in human prion disease.. Neurobiol Dis.

[8] Hayashi M, Arai N, Satoh J, Suzuki H, Katayama K (2002). Neurodegenerative mechanisms in subacute sclerosing panencephalitis.. J Child Neurol.

[9] Nunomura A, Chiba S, Kosaka K, Takeda A, Castellani RJ (2002). Neuronal RNA oxidation is a prominent feature of dementia with Lewy bodies.. Neuroreport.

[10] Petersen RB, Siedlak SL, Lee HG, Kim YS, Nunomura A (2005). Redox metals and oxidative abnormalities in human prion diseases.. Acta Neuropathol (Berl).

[11] Shan X, Lin CL (2006). Quantification of oxidized RNAs in Alzheimer's disease.. Neurobiol Aging.

[12] Shan X, Tashiro H, Lin CL (2003). The identification and characterization of oxidized RNAs in Alzheimer's disease.. J Neurosci.

[13] Shan X, Chang Y, Lin CL (2007). Messenger RNA oxidation is an early event preceding cell death and causes reduced protein expression.. FASEB J.

[14] Tanaka M, Chock PB, Stadtman ER (2007). Oxidized messenger RNA induces translation errors.. Proc Natl Acad Sci U S A.

[15] Ding Q, Markesbery WR, Chen Q, Li F, Keller JN (2005). Ribosome dysfunction is an early event in Alzheimer's disease.. J Neurosci.

[16] Honda K, Smith MA, Zhu X, Baus D, Merrick WC (2005). Ribosomal RNA in Alzheimer disease is oxidized by bound redox-active iron.. J Biol Chem.

[17] Nunomura A, Chiba S, Lippa CF, Cras P, Kalaria RN (2004). Neuronal RNA oxidation is a prominent feature of familial Alzheimer's disease.. Neurobiol Dis.

[18] Nunomura A, Perry G, Aliev G, Hirai K, Takeda A (2001). Oxidative damage is the earliest event in Alzheimer disease.. J Neuropathol Exp Neurol.

[19] Boillee S, Vande Velde C, Cleveland DW (2006). ALS: a disease of motor neurons and their nonneuronal neighbors.. Neuron.

[20] Julien JP, Kriz J (2006). Transgenic mouse models of amyotrophic lateral sclerosis.. Biochim Biophys Acta.

[21] Shefner JM, Reaume AG, Flood DG, Scott RW, Kowall NW (1999). Mice lacking cytosolic copper/zinc superoxide dismutase display a distinctive motor axonopathy.. Neurology.

[22] Lobsiger CS, Cleveland DW (2007). Glial cells as intrinsic components of non-cell-autonomous neurodegenerative disease.. Nat Neurosci.

[23] Khatri P, Draghici S, Ostermeier GC, Krawetz SA (2002). Profiling gene expression using onto-express.. Genomics.

[24] Graf M, Ecker D, Horowski R, Kramer B, Riederer P (2005). High dose vitamin E therapy in amyotrophic lateral sclerosis as add-on therapy to riluzole: results of a placebo-controlled double-blind study.. J Neural Transm.

[25] Andrus PK, Fleck TJ, Gurney ME, Hall ED (1998). Protein oxidative damage in a transgenic mouse model of familial amyotrophic lateral sclerosis.. J Neurochem.

[26] Hall ED, Andrus PK, Oostveen JA, Fleck TJ, Gurney ME (1998). Relationship of oxygen radical-induced lipid peroxidative damage to disease onset and progression in a transgenic model of familial ALS.. J Neurosci Res.

[27] Liu D, Wen J, Liu J, Li L (1999). The roles of free radicals in amyotrophic lateral sclerosis: reactive oxygen species and elevated oxidation of protein, DNA, and membrane phospholipids.. FASEB J.

[28] Gurney ME, Cutting FB, Zhai P, Doble A, Taylor CP (1996). Benefit of vitamin E, riluzole, and gabapentin in a transgenic model of familial amyotrophic lateral sclerosis.. Ann Neurol.

[29] Gard AL, Solodushko VG, Waeg G, Majic T (2001). 4-Hydroxynonenal, a lipid peroxidation byproduct of spinal cord injury, is cytotoxic for oligodendrocyte progenitors and inhibits their responsiveness to PDGF.. Microsc Res Tech.

[30] Juurlink BH, Thorburne SK, Hertz L (1998). Peroxide-scavenging deficit underlies oligodendrocyte susceptibility to oxidative stress.. Glia.

[31] Wong PC, Pardo CA, Borchelt DR, Lee MK, Copeland NG (1995). An adverse property of a familial ALS-linked SOD1 mutation causes motor neuron disease characterized by vacuolar degeneration of mitochondria.. Neuron.

[32] Jonsson PA, Ernhill K, Andersen PM, Bergemalm D, Brannstrom T (2004). Minute quantities of misfolded mutant superoxide dismutase-1 cause amyotrophic lateral sclerosis.. Brain.

[33] Williamson TL, Cleveland DW (1999). Slowing of axonal transport is a very early event in the toxicity of ALS-linked SOD1 mutants to motor neurons.. Nat Neurosci.

[34] Wang J, Xu G, Gonzales V, Coonfield M, Fromholt D (2002). Fibrillar inclusions and motor neuron degeneration in transgenic mice expressing superoxide dismutase 1 with a disrupted copper-binding site.. Neurobiol Dis.

[35] Ferri A, Cozzolino M, Crosio C, Nencini M, Casciati A (2006). Familial ALS-superoxide dismutases associate with mitochondria and shift their redox potentials.. Proc Natl Acad Sci U S A.

[36] LaMonte BH, Wallace KE, Holloway BA, Shelly SS, Ascano J (2002). Disruption of dynein/dynactin inhibits axonal transport in motor neurons causing late-onset progressive degeneration.. Neuron.

[37] Puls I, Jonnakuty C, LaMonte BH, Holzbaur EL, Tokito M (2003). Mutant dynactin in motor neuron disease.. Nat Genet.

[38] Nishimura AL, Mitne-Neto M, Silva HC, Richieri-Costa A, Middleton S (2004). A mutation in the vesicle-trafficking protein VAPB causes late-onset spinal muscular atrophy and amyotrophic lateral sclerosis.. Am J Hum Genet.

[39] Weir ML, Klip A, Trimble WS (1998). Identification of a human homologue of the vesicle-associated membrane protein (VAMP)-associated protein of 33 kDa (VAP-33): a broadly expressed protein that binds to VAMP.. Biochem J.

[40] Xiao S, McLean J, Robertson J (2006). Neuronal intermediate filaments and ALS: a new look at an old question.. Biochim Biophys Acta.

[41] Kabashi E, Agar JN, Taylor DM, Minotti S, Durham HD (2004). Focal dysfunction of the proteasome: a pathogenic factor in a mouse model of amyotrophic lateral sclerosis.. J Neurochem.

[42] Tummala H, Jung C, Tiwari A, Higgins CM, Hayward LJ (2005). Inhibition of chaperone activity is a shared property of several Cu,Zn-superoxide dismutase mutants that cause amyotrophic lateral sclerosis.. J Biol Chem.

[43] Jung C, Higgins CM, Xu Z (2002). Mitochondrial electron transport chain complex dysfunction in a transgenic mouse model for amyotrophic lateral sclerosis.. J Neurochem.

[44] Borthwick GM, Johnson MA, Ince PG, Shaw PJ, Turnbull DM (1999). Mitochondrial enzyme activity in amyotrophic lateral sclerosis: implications for the role of mitochondria in neuronal cell death.. Ann Neurol.

[45] Browne SE, Yang L, DiMauro JP, Fuller SW, Licata SC (2006). Bioenergetic abnormalities in discrete cerebral motor pathways presage spinal cord pathology in the G93A SOD1 mouse model of ALS.. Neurobiol Dis.

[46] Smith AP, Lee NM (2007). Role of zinc in ALS.. Amyotroph Lateral Scler.

[47] Gong YH, Elliott JL (2000). Metallothionein expression is altered in a transgenic murine model of familial amyotrophic lateral sclerosis.. Exp Neurol.

[48] Nagano S, Satoh M, Sumi H, Fujimura H, Tohyama C (2001). Reduction of metallothioneins promotes the disease expression of familial amyotrophic lateral sclerosis mice in a dose-dependent manner.. Eur J Neurosci.

[49] Collard JF, Cote F, Julien JP (1995). Defective axonal transport in a transgenic mouse model of amyotrophic lateral sclerosis.. Nature.

[50] Banik NL, Hogan EL, Hsu CY (1987). The multimolecular cascade of spinal cord injury. Studies on prostanoids, calcium, and proteinases.. Neurochem Pathol.

[51] Ekegren T, Hanrieder J, Aquilonius SM, Bergquist J (2006). Focused proteomics in post-mortem human spinal cord.. J Proteome Res.

[52] Yamamoto Y, Yoshikawa H, Nagano S, Kondoh G, Sadahiro S (1999). Myelin-associated oligodendrocytic basic protein is essential for normal arrangement of the radial component in central nervous system myelin.. Eur J Neurosci.

[53] Ascherio A, Weisskopf MG, O'Reilly EJ, Jacobs EJ, McCullough ML (2005). Vitamin E intake and risk of amyotrophic lateral sclerosis.. Ann Neurol.

[54] Guo H, Lai L, Butchbach ME, Lin CL (2002). Human glioma cells and undifferentiated primary astrocytes that express aberrant EAAT2 mRNA inhibit normal EAAT2 protein expression and prevent cell death.. Mol Cell Neurosci.

